# Green Waste Compost Impacts Microbial Functions Related to Carbohydrate Use and Active Dispersal in Plant Pathogen-Infested Soil

**DOI:** 10.1007/s00248-024-02361-8

**Published:** 2024-02-17

**Authors:** Nicholas R. LeBlanc, Fiona C. Harrigian

**Affiliations:** United States Department of Agriculture, Agricultural Research Service, Crop Improvement and Protection Research Unit, 1636 E. Alisal St, Salinas, CA 93905 USA

**Keywords:** Soil health, *Fusarium oxysporum*, Nanopore sequencing, Biological control

## Abstract

The effects of compost on physical and chemical characteristics of soil are well-studied but impacts on soil microbiomes are poorly understood. This research tested effects of green waste compost on bacterial communities in soil infested with the plant pathogen *Fusarium oxysporum*. Compost was added to pathogen-infested soil and maintained in mesocosms in a greenhouse experiment and replicated growth chamber experiments. Bacteria and *F. oxysporum* abundance were quantified using quantitative PCR. Taxonomic and functional characteristics of bacterial communities were measured using shotgun metagenome sequencing. Compost significantly increased bacterial abundance 8 weeks after amendment in one experiment. Compost increased concentrations of chemical characteristics of soil, including phosphorus, potassium, organic matter, and pH. In all experiments, compost significantly reduced abundance of *F*. *oxysporum* and altered the taxonomic composition of soil bacterial communities. Sixteen bacterial genera were significantly increased from compost in every experiment, potentially playing a role in pathogen suppression. In all experiments, there was a consistent negative effect of compost on functions related to carbohydrate use and a positive effect on bacteria with flagella. Results from this work demonstrate that compost can reduce the abundance of soilborne plant pathogens and raise questions about the role of microbes in plant pathogen suppression.

**Supplementary Information:**

The online version contains supplementary material available at 10.1007/s00248-024-02361-8.

## Introduction

Soil microbiomes mediate multiple processes that feedback on crop growth and the long-term maintenance of soil health in agricultural ecosystems [1, 2]. A primary taxonomic component of soil microbiomes is bacteria; they regulate transformation and availability of primary plant nutrients like nitrogen (N), phosphorus (P), and potassium (K) as well as decomposition and soil organic matter accumulation [3–5]. Interactions among bacteria, other soil microorganisms, and plant roots can also influence plant physiology and increase crop tolerance to abiotic and biotic stresses [6, 7]. Therefore, understanding impacts of common agricultural practices on soil microbiomes is needed to improve crop health and reduce the environmental footprint of agriculture [1].

Compost generated from plant or animal byproducts is a widely used soil amendment that impacts soil characteristics that are beneficial to agriculture and the broader environment [8]. Incorporation of compost into soil can increase growth-limiting plant nutrients thereby reducing reliance on synthetic fertilizers [9, 10]. Studies have also highlighted the positive effects of compost on soil organic matter and physical characteristics of soil that reduce erosion and improve water infiltration [9, 11, 12]. Long-term positive effects on soil carbon sequestration and reduction of CO_2_ emissions by recycling agricultural waste through composting further demonstrate the potential for compost to play a role in climate change mitigation efforts in agriculture [13–15].

As well as soil chemical properties, compost can alter the activity of soil microorganisms that play pivotal roles in agriculture. Soilborne plant pathogens are a common constraint in agriculture where they reduce crop quality and yield [16]. Fungal pathogens in the *Fusarium oxysporum* species-complex are especially problematic due to their cosmopolitan distribution and ability to live as saprotrophs in soil [17]. Multiple studies have shown green waste compost (i.e., compost generated from plant material) or green waste compost mixed with composted manure can reduce disease on lettuce and other crops caused by *F. oxysporum*, though the level of disease suppression can be variable across experiments and studies [18, 19]. More recent research has shown that green waste compost also reduces the abundance of the pathogen itself in soil [20]. While specific mechanisms of compost-based disease and pathogen suppression are poorly understood, microorganisms in soil are thought to play a central role in this process [21, 22].

To understand how compost suppresses plant pathogens, it is important to know how compost effects other soil microorganisms. As one might expect, adding various green waste composts that can increase organic matter (OM) tends to increase microbial biomass [23, 24]. Green waste compost often significantly alters microbial community composition regardless of whether microbial diversity and richness increase or not [25, 26]. In addition to the changes in microbial biomass and community composition, green waste compost has potential to alter microbial functional characteristics. For example, prior work has shown green waste compost increases how fast substrates are used by bacterial communities [24]. However, the effects of green waste compost on other functions that may impact microbial or plant–microbe interactions in soil are largely unknown.

The goal of this study was to evaluate the effects of compost on bacteria in soil infested with the common soilborne plant pathogen *F. oxysporum*. We hypothesized that compost would increase bacterial abundance and decrease *F. oxysporum* abundance, while altering taxonomic and functional characteristics of soil bacterial communities. Outcomes of this research confirm the suppressive effects of green waste compost toward fungal plant pathogens. This study also provides novel insight into impacts of compost on taxonomic and functional characteristics of bacterial communities as primary components of microbiomes in agricultural soil.

## Materials and Methods

### Compost and Plant Pathogen-Infested Soil

Commercially available green waste compost generated from plant material (garden prunings, food products, and vegetable trimmings) was used in all experiments (Cedar Grove Composting, WA, USA). The compost had 53% organic matter and a carbon to nitrogen ratio of 18. Bagged compost was maintained for about 5 months at ambient temperature between use in the initial greenhouse experiment and subsequent replicated growth chamber experiments.

Field soil was artificially infested with *Fusarium oxysporum* f. sp. *lactucae*, the causal agent of the disease fusarium wilt of lettuce. Field soil was from a research plot at the USDA-ARS Crop Improvement and Protection Research Unit in Salinas, CA. Pathogen-infested sand inoculum was generated from *F. oxysporum* using previously described methods [27]. Pathogen colony-forming units (CFUs) in infested and non-infested (negative control) sand were measured with semi-selective Komada’s media, prior to use in experiments [28].

### Greenhouse Experiment

Soil mesocosms were established to test the effect of compost on bacterial communities in soil infested with the soilborne pathogen *F. oxysporum*. Pathogen-infested sand inoculum was added to soil for an approximate 1.6 × 10^4^ *F. oxysporum* CFUs/g of soil (5% sand by weight). Negative control treatments included sterile sand without the pathogen present. Compost was added at 5% and 10% to pathogen-infested soil (by weight). Soil was added to 3-in pots (200 g/pot) and soil moisture was adjusted weekly to field capacity (20% soil moisture) to simulate irrigation events. Soil samples were collected 3 and 8 weeks after amendment. Each 500-mg soil sample was randomly selected within individual pots. Each treatment had three replicates and mesocosms were maintained under greenhouse conditions in a completely randomized design. Average temperature in the greenhouse during this experiment (± SD) was 23.1 ± 7.9 °C and average relative humidity (± SD) was 68.9 ± 19.6% based on hourly data-logger readings.

### Growth Chamber Experiments

Two replicated chamber experiments were conducted at different times to evaluate reproducibility of the effects of compost under the same experimental conditions. Soil was infested with *F. oxysporum* using the same proportion of sand as previously described but at a higher concentration of the pathogen (2 × 10^5^ CFUs/gram of soil). Compost was added to soil at a single 10% rate and aliquoted into pots with three replicates per treatment. Mesocosms were maintained in a completely randomized design in a growth chamber set with a 16-h light (28 °C) and 8-h dark (20 °C) daily cycle. Soil moisture was adjusted weekly as described above. After 3 weeks, 500-mg soil samples were collected from each treatment replicate for DNA extractions as described above.

### Soil Sampling and Processing

Soil DNA was extracted using the DNeasy PowerSoil Pro Kit (Qiagen, MD, USA). Initial bead beating of soil was conducted using three 30-s pulses on a Mini-BeadBeater-16 (BioSpec Products, OK, USA). Extracted DNA was quantified using a NanoDrop ONE spectrophotometer (Thermo Fisher Scientific, MA, USA). Soil chemical characteristics were measured from the greenhouse experiment at the University of Idaho Analytical Sciences Laboratory (https://www.uidaho.edu/cals/analytical-sciences-laboratory).

### Quantitative PCR

Bacterial DNA concentration was measured using qPCR as a proxy for bacterial biomass with universal bacterial 16S rRNA primers Eub338F (5′-ACTCCTACGGGAGGCAGCAG-3′) [29] and Eub518R (5′-ATTACCGCGGCTGCTGG-3′) [30]. The abundance of *F. oxysporum* f. sp. *lactucae* was measured using published primers hani3′ (5′-CCCTCCAACATTCAACAACTG-3′) and hanilatt3rev (5′-ATTCACTGTACACCAACCTTTT-3′) [31, 32]. All reactions were conducted using equipment and reagents as previously described [33]. Reactions were run with an initial denaturing step of 98 °C (3 m), followed by 35 cycles of 98 °C (10 s), annealing/extension (30 s), and plate read. Quantification of bacterial abundance used an annealing temperature of 57 °C and *F. oxysporum* 60 °C. Standard curves (10 ng/µl to 1 × 10^−4^ ng/µl) were generated using genomic DNA from an unidentified *Stenotrophomonas* sp. for the 16S rRNA assay and *F. oxysporum* f.sp. *lactucae* NRRL26844 for the *F. oxysporum* assay. All plates included a positive control standard and negative control wells. Quantification of *F. oxysporum* f. sp. *lactucae* abundance also included DNA from the tomato pathogen *F. oxysporum* f. sp. *lycopersici* NRRL26380 as a second negative control. A summary of qPCR results are in Supplementary Table 1.

### Nanopore Sequencing and Bioinformatics

Soil microbiomes were characterized using nanopore sequencing. Libraries were prepared and sequenced on a MinION sequencer as previously described (Oxford Nanopore Technologies, Oxford, UK) [33]. Sequences less than 500 bp were filtered from metagenome data using NanoFilt v2.7.1 [34]. Remaining sequences were demultiplexed by barcode using Porechop v0.2.3 (https://github.com/rrwick/Porechop). Sequence data were classified to bacterial genera and higher taxonomies using the Kraken2 standard database downloaded 4/4/2022 [35]. Metagenome sequence data were also classified to functions using MEGAN v6.21.5 [36] and DIAMOND v2.0.6 [37] as previously described [33]. Functions were analyzed at the highest resolution of the SEED sub-system implemented in MEGAN [38]. Data were filtered to only include bacterial data and to remove non-microbial data. Sequence data were normalized to account for variation in sequence depth by rarefying samples based on the sample with the fewest number of classified sequences. Sequencing output and classification are summarized in Supplementary Table 2.

### Statistical Analyses

Statistical analyses were conducted in R v4.1.2 [39]. Analysis of variance (ANOVA) followed by the Tukey Honestly Significant Differences (TukeyHSD) post-hoc test were used for experiments with more than two treatments. *T*-tests were used for experiments with two treatments. Model assumptions were evaluated using Levene’s test to determine homogeneity of variance across groups, implemented in the package car v3.1–2 [40], and quantile–quantile plots to determine if data followed normal distributions. Data violating assumptions were further tested using the non-parametric Kruskal–Wallis rank sum test followed by Dunn’s Test for post-hoc comparisons. Mixed effect models were employed to test for an effect of time on measured variables since data from different timepoints in the greenhouse experiment were collected from the same pot (i.e., non-independent samples). Models compared compost treatments, timepoint (three versus eight weeks), and interaction between these terms as fixed effects and sampled pot as a random effect. Tests and post-hoc comparisons were conducted using the nlme v3.1–162 [41] and emmeans v1.9.0 [42] packages by aggregating across time and compost treatments. Bacterial diversity was calculated as Shannon’s Index (*H′*). Variation in bacterial community composition was evaluated based on Bray–Curtis distances using nonmetric multidimensional scaling (NMDS) with the package vegan v2.5.7 [43]. Soil chemical characteristics were fit to ordination data using the function envfit. Permutational multivariate analysis of variance using distance matrices (PERMANOVA) implemented in the vegan v2.5.7 package [43] was used to test for an effect of compost on bacterial community composition. The PERMDISP test, implemented with the betadisper function in vegan, was used to evaluate if differences among treatments were due to within group variability, rather than differences in group means. The effect of compost on individual bacterial taxa and functions was tested using DESeq2 v1.34.0. [44]. Pairwise comparisons were made using the Wald test between negative controls and 10% compost treatments for consistency among experiments. The false discovery rate (FDR) correction was used to account for multiple comparisons [44]. Low abundance taxa or functions represented by fewer than 100 sequences were filtered prior to DESeq2 analysis. Trees were generated using PhyloT v2 (https://phylot.biobyte.de) and plotted using the Interactive Tree of Life (iTOL) v6 (https://itol.embl.de) [45]. Data were plotted with ggplot2 v3.3.5 [46].

## Results

### Effect of Compost on Chemical and Microbial Characteristics of Soil

In the initial greenhouse experiment, 3 weeks following compost application, compost applied at 10% significantly reduced *Fusarium oxysporum* abundance and increased bacterial abundance compared to the negative control, but neither the 5% nor the 10% compost application led to significantly higher bacterial abundance (Table 1). Bacterial diversity was slightly reduced in soils with compost, but not significantly. Richness was not higher or lower 3 weeks after the application of compost. Eight weeks following compost application, compost applied at 10% significantly increased bacterial abundance and compost at both 5% and 10% significantly reduced *Fusarium oxysporum* abundance. Bacterial richness trended slightly higher when compost was applied but was not significantly higher. Bacterial diversity did not exhibit any trend, higher or lower (Table 1). In addition to evaluating the effect of compost, the effect of time was also measured in a mixed model with post-hoc tests. When all samples taken at each timepoint were measured, regardless of treatment *Fusarium* abundance significantly decreased by 62.57% by week 8 (T ratio = 6.337, *P* < 0.0001, Supplementary Table 5), bacterial abundance significantly increased by 43.89% by week 8 (T ratio =  − 3.751, *P* < 0.01), and bacterial diversity significantly increased by 6.72% by week 8 (T ratio =  − 9.222, *P* < 0.0001). Bacterial richness increased by 1.61%, which was not significantly different from week 3 (T ratio =  − 1.228, *P* = 0.2477). When examining the effects of compost regardless of time, post-hoc tests agreed with the previously conducted ANOVAs (Table 1, Supplementary Table 5).

**Table 1:** Bacterial and *Fusarium oxysporum* abundance, bacterial diversity, and richness 3 and 8 weeks following application of compost (5% and 10%) to soil mesocosms in the greenhouse. Means with same letter in same column are not significantly different (*P* < 0.05) based on analysis of variance (ANOVA). Characteristics with “*” were found to violate assumptions of ANOVA and non-parametric Kruskal–Wallis rank sum test was used. SD, standard deviation. Each treatment had three replicates. Units for abundance measurements are ng/µl. Bacterial diversity was measured based on the Shannon Index (*H*′). Bacterial diversity and richness are based on genus level classification

	Bacterial abundance	Bacterial diversity*	Bacterial richness	*Fusarium* abundance
Week 3				
Control (Mean ± SD)	1.64 ± 0.2 a	4.99 ± 0.2 a	1320 ± 72.5 a	0.0136 ± 0.003 a
Compost (5%) (Mean ± SD)	2.57 ± 0.5 a	4.76 ± 0.06 a	1310 ± 24.8 a	0.00842 ± 0.0004 ab
Compost (10%) (Mean ± SD)	2.58 ± 0.7 a	4.78 ± 0.02 a	1340 ± 18.3 a	0.00506 ± 0.003 b
Week 8				
Control (Mean ± SD)	2.26 ± 0.4 a	5.20 ± 0.03 a	1320 ± 23.4 a	0.00711 ± 0.003 a
Compost (5%) (Mean ± SD)	3.51 ± 0.6 ab	5.15 ± 0.06 a	1360 ± 22.9 a	0.00211 ± 0.0004 b
Compost (10%) (Mean ± SD)	4.0068 ± 0.8 b	5.16 ± 0.03 a	1360 ± 28.05 a	0.000935 ± 0.0006 b

At the culmination of the greenhouse experiment, compost significantly increased the concentration of K, P, and OM. Furthermore, 10% added compost significantly increased the concentration of K, P, and OM even when compared to soils with 5% added compost. pH was significantly increased in the soil with 10% compost added. N concentration was not significantly altered by compost at either 5% or 10% application rates (Table 2).

**Table 2:** Soil chemical characteristics at 8 weeks following application of compost (5% and 10%) to soil mesocosms. K, potassium (ppm); P, phosphorus (ppm); N, nitrogen (ppm); OM, organic matter (%); means with same letter in same column are not significantly different using *P* < 0.05 based on analysis of variance (ANOVA). Characteristics with “*” were found to violate assumptions of ANOVA, and non-parametric Kruskal–Wallis rank sum test was used. SD, standard deviation. Each treatment had three replicates

	K	P	N	OM	pH*
Control (Mean ± SD)	180 ± 17.3 a	52.3 ± 0.5 a	19.0 ± 1.0 a	1.43 ± 0.1 a	7.50 ± 0.0 a
Compost (5%) (Mean ± SD)	263 ± 15.3 b	61.3 ± 3.2 b	18.0 ± 1.0 a	2.27 ± 0.1 b	7.53 ± 0.1 ab
Compost (10%) (Mean ± SD)	407 ± 37.9 c	69.7 ± 3.8 c	19.7 ± 1.2 a	2.87 ± 0.2 c	7.63 ± 0.1 b

In the first growth chamber experiment, bacterial diversity and bacterial richness were significantly higher in soil with the addition of 10% compost compared to control soil. *Fusarium oxysporum* abundance was significantly suppressed by the addition of 10% compost. Bacterial abundance was not significantly different when compost had been added. In the second growth chamber experiment, *Fusarium oxysporum* abundance was also significantly suppressed by the 10% compost addition just as it had in the first growth chamber experiment. However, bacterial abundance, richness, and diversity were not significantly altered with the addition of 10% compost (Table 3).

**Table 3:** Bacterial and *Fusarium oxysporum* abundance, bacterial diversity, and richness following application of compost at 10% to soil in growth chamber experiments. Means with same letter in same row are not significantly different (*P* < 0.05) based on analysis of variance (ANOVA). SD, standard deviation. Each treatment had three replicates. Units for abundance measurements are ng/µl. Bacterial diversity was measured based on the Shannon Index (*H*′). Bacterial diversity and richness are based on genus level classification

	Bacterial abundance	Bacterial diversity	Bacterial richness	*Fusarium* abundance
Growth chamber experiment 1				
Control (Mean ± SD)	1.98 ± 0.6 a	5.06 ± 0.07 a	1460 ± 41.5 a	0.109 ± 0.03 a
Compost (10%) (Mean ± SD)	1.46 ± 0.6 a	5.24 ± 0.03 b	1540 ± 13.7 b	0.0392 ± 0.007 b
Growth chamber experiment 2				
Control (Mean ± SD)	1.70 ± 0.5 a	5.24 ± 0.05 a	1540 ± 6.2 a	0.0674 ± 0.02 a
Compost (10%) (Mean ± SD)	1.11 ± 0.4 a	5.24 ± 0.09 a	1540 ± 25.3 a	0.0314 ± 0.01 b

### Effect of Compost on Bacterial Community Composition

**Fig. 1:**
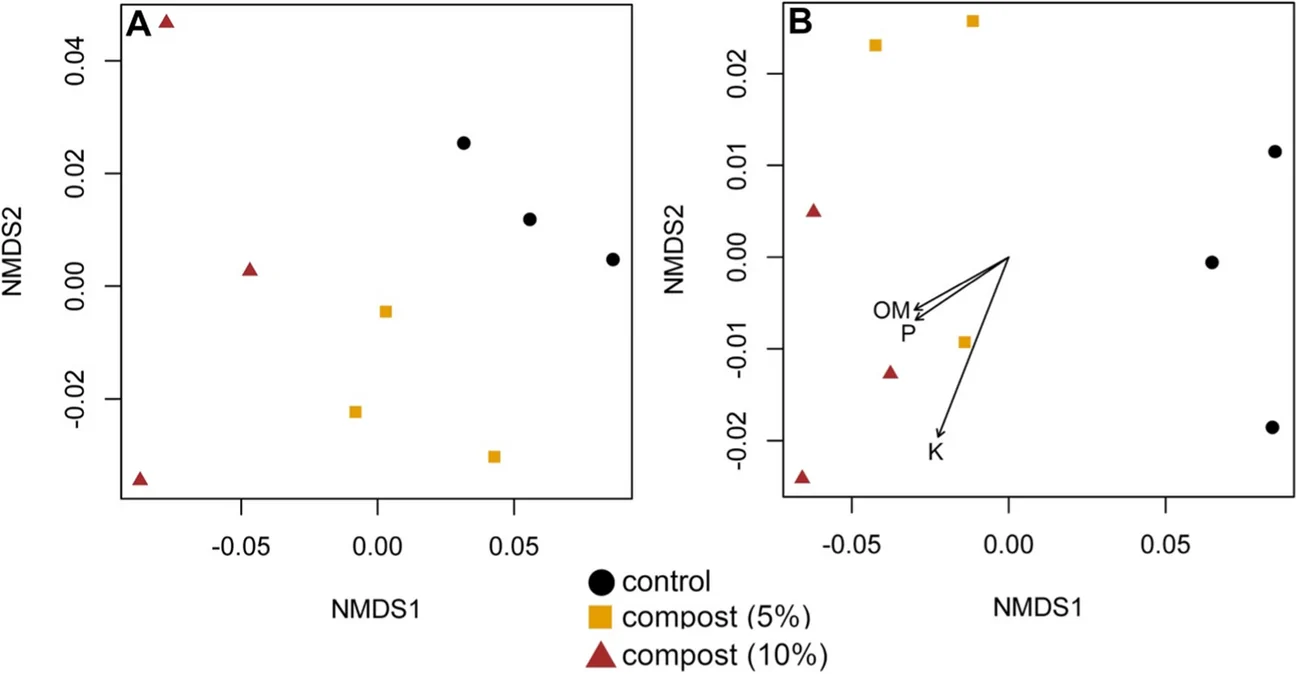
Ordination plot showing variation in bacterial community composition from greenhouse experiment based on nonmetric multidimensional scaling (NMDS). Sub-plot **A** shows data from 3 weeks after amending soil with compost, and sub-plot **B** shows data from 8 weeks after amendment. Axes in sub-plot **B** show soil chemical characteristics that are significantly (*P* < 0.05) correlated with NMDS ordination axes (OM, organic matter; P, phosphorus; K, potassium)

Ordination plots of bacterial community composition showed a clear separation among low and high compost treatments and control soils at 3 and 8 weeks post amendment in the initial greenhouse experiment (Fig. 1A, B). Samples from control soil containing sand and no inoculum from the pathogen *F. oxysporum* clustered with the primary control treatment (Supplementary Fig. 1). Soil OM, P, and K were significantly correlated with NMDS ordination axes at the termination of the experiment (Fig. 1B). In addition, PERMANOVA tests demonstrated compost treatments explained a significant amount of variation in bacterial community composition at 3 (*F* = 5.792, *P* < 0.01) and 8 weeks (*F* = 8.5003, *P* < 0.01) after amendment. The PERMDISP test was not significant for 3- (*F* = 0.0727, *P* = 0.9306) and 8-week timepoints (*F* = 1.2267, *P* = 0.3576), indicating that significant differences were not due to variability within groups.

Analysis of data from replicated growth chamber experiments showed samples clustered based on control and compost treated soils (Supplementary Fig. 2). Based on PERMANOVA tests, there was no significant effect of compost application on bacterial community composition in the first experiment (*F* = 17.172, *P* < 0.1) and second growth chamber experiment (*F* = 6.1158, *P* < 0.1). Analysis of data aggregated across the two replicated experiments, treating experiments as blocks (i.e., strata), showed that there was a significant effect of compost on bacterial community composition based on PERMANOVA (*F* = 11.864, *P* < 0.01). There was no significant effect of within group variability based on PERMDISP (*F* = 0.0011, *P* = 0.9745).

**Fig. 2:**
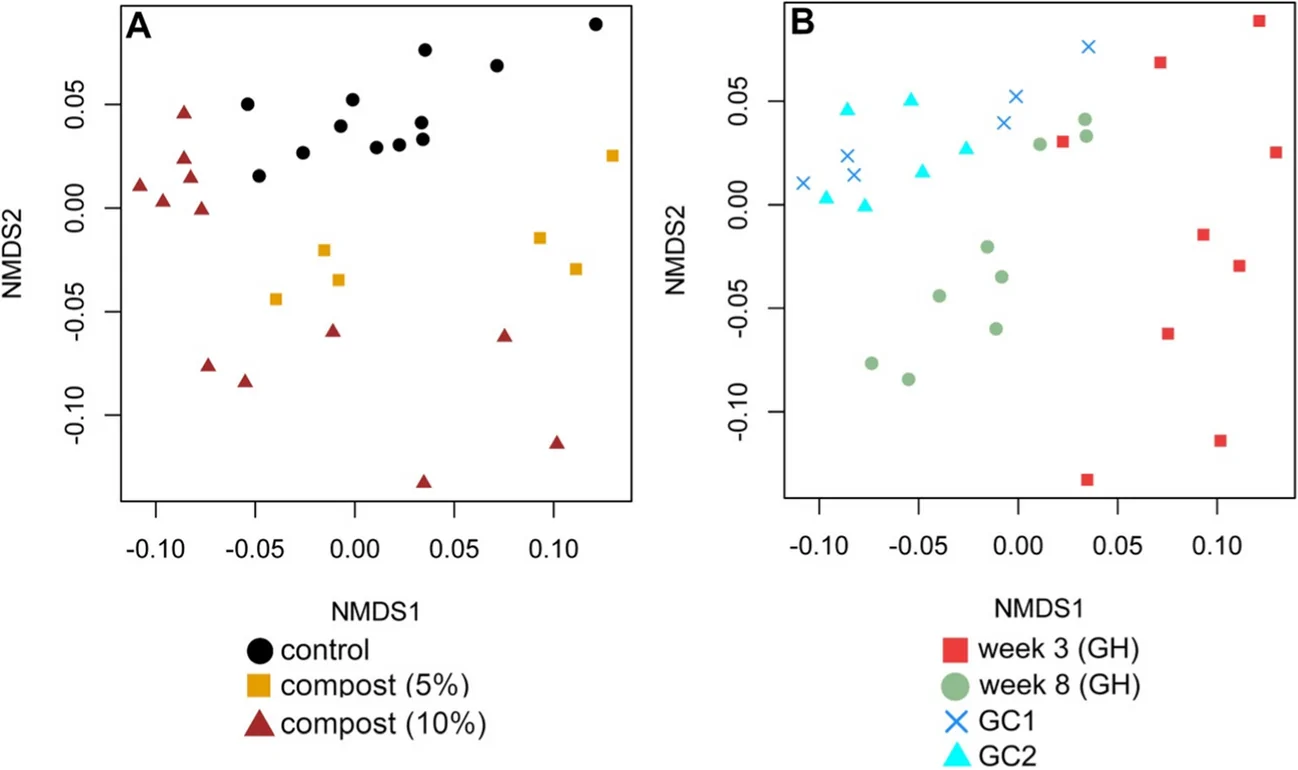
Ordination plot showing variation in bacterial community composition across all experiments and environments based on nonmetric multidimensional scaling (NMDS). Sub-plot **A** shows differences among experimental treatments. Sub-plot **B** shows differences among experiments and environments (GH, greenhouse experiments; GC1, first growth chamber experiment; and GC2, second growth chamber experiment)

Further analysis of data from all experiments and environments showed samples in compost-treated samples clustered away from fallow soils in ordination space. In addition, samples are clustered by timepoints in the greenhouse experiment and by separate environments (greenhouse versus growth chamber) (Fig. 2). Application of PERMANOVA and treating separate experiments as blocks showed a significant effect of compost on bacterial community composition (*F* = 4.7693, *P* < 0.001) across this aggregated dataset. Based on the PERMDISP test, there was evidence that differences were also attributed to within group variability (*F* = 13.65, *P* < 0.001).

### Effect of Compost on Taxa and Functional Traits

Pairwise comparisons between control and 10% compost application treatments were conducted for all timepoints and experiments to identify if specific bacterial taxa or functions were consistently impacted by compost application among experiments and environments. Eighty-nine bacterial genera were differentially abundant in the greenhouse experiment at the 3-week timepoint (66 significantly higher in compost amended soil and 23 significantly lower). At the 8-week timepoint of the same experiment, there were 185 differentially abundant taxa (130 were significantly higher in compost amended soil and 55 significantly lower). Seventy-three taxa were significantly differentially abundant at both timepoints, all showing the same positive or negative response to compost application at 3 and 8 weeks (Supplementary Table 3).

In the first growth chamber experiment, 132 bacterial genera were differentially abundant (126 significantly increased from compost and 6 significantly decreased). In the second replicate growth chamber experiment, 83 bacterial taxa were differentially abundant (59 significantly increased from compost and 24 significantly decreased). Comparisons between data from the two experiments showed that 64 taxa were differentially abundant in both replicate growth chamber experiments. All these taxa showed the same positive or negative response to compost, except for the genus *Modestobacter* which significantly decreased from compost in the first experiment and increased from compost in the second experiment. Of the 64 differentially abundant taxa, 32 were significantly affected in at least one of the two timepoints in the greenhouse experiment. Twenty-two of these taxa showed the same positive or negative response to compost application in all experiments. Those showing consistent negative responses were within the families *Bacillaceae* (*Metabacillus*, *Fictibacillus*, and *Priestia*), *Paenibacillaceae* (*Paenibacillus*), *Micrococcaceae* (*Pseudoarthrobacter*), and *Rubrobacteriaceae* (*Rubrobacter*). Genera showing consistent increases from compost were in the families *Flavobacteriaceae* (*Muricauda*), *Caulobacteraceae* (*Phenylbacterium* and *Caulobacter*), *Hyphomicrobiales incertae sedis* (*Terrihabitans*), *Devosiaceae* (*Devosia*, *Paradevosia*, and *Youhaiella*), *Nocardioidaceae* (*Rhodococcus* and *Aeromicrobium*), *Thermoactinomycetaceae*(*Thermoactinomyces*), *Paenibacillaceae* (*Thermobacillus*), *Planococcaceae* (*Ureibacillus*), and *Bacillaceae* (*Aeribacillus*, *Geobacillus*, *Parageobacillus*, and *Weizmannia*) (Supplementary Table 3; Supplementary Fig. 3).

Functional analysis identified 15 differentially abundant functions 3 weeks after amendment in the greenhouse experiment, seven higher in control soil and eight higher in compost treatment. At 8 weeks, there were 89 differentially abundant functions, 36 higher in control soil and 53 higher in compost treated soil. Twelve functions were significantly affected at both 3 and 8 weeks. Comparison of data from both growth chamber experiments showed a total of 77 differentially abundant functions in the first and second experiment. Thirty of these functions showed consistent responses to compost application in both replicate growth chamber experiments, six higher in control soil and 15 higher in compost treated soil (Supplementary Table 4).

**Fig. 3:**
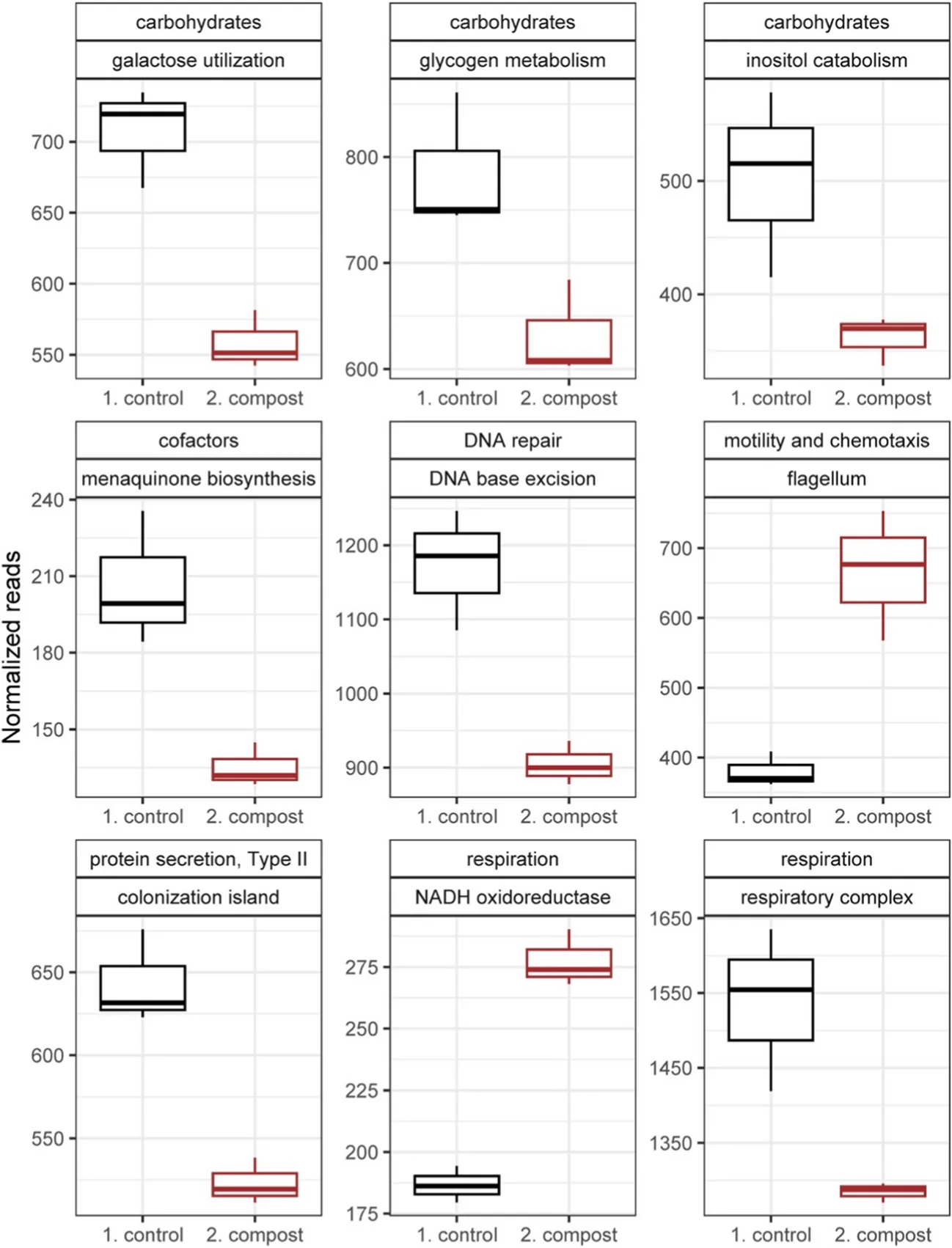
Boxplots showing bacterial functions significantly influenced by compost application in greenhouse and replicated growth chamber experiments. The y-axis shows normalized reads for a given function and x-axis shows negative control and compost amended soil treatments. Differences are based on DESeq2. Each treatment has three replicates. Plot titles show high order SEED classification (e.g., carbohydrates) and specific functions (e.g., galactose utilization). The following titles are abbreviated to improve clarity: DNA base excision = DNA repair base excision, menaqionone biosynthesis = menaquinone biosynthesis from chorismate via 1,4-dihydroxy-6-naphthoate, NADH oxidoreductase = NADH ubiquinone oxidoreductase vs. multi-subunit cation antiporter, respiratory complex = respiratory complex I, colonization island = widespread colonization island. All differences between treatments are statistically significant (*P* < 0.05) with raw data summarized in Supplementary Table 4. Presented data are from the final timepoint of the greenhouse experiment. A complete set of figures for all timepoints and experiments are in Supplementary Fig. 4

Further comparison of data from both environments showed nine functions were significantly affected in both growth chamber experiments and at least one timepoint from the greenhouse experiment. Three functions in the higher-level SEED classification “carbohydrates” were significantly higher in control soil compared to compost treated soil, including “galactose utilization,” “glycogen metabolism,” and “inositol catabolism.” Additional functions that were higher in control soil were “menaquinone biosynthesis via chorismate 1,4-dihydroxy-6-naphthoate,” “DNA repair base excision,” “widespread colonization island,” and “respiratory complex I.” The two functions that were significantly higher in compost-treated soil were “NADH ubiquinone oxidoreductase vs. multi-subunit cation antiporter” and “flagellum” (Fig. 3, Supplementary Fig. 4, and Supplementary Table 4).

## Discussion

Compost is a widely used soil amendment, yet its effects on soil microbiomes are still poorly understood. In this study, the effects of green waste compost were evaluated in independent soil mesocosm experiments. Consistent with prior research, compost increased the concentration of multiple soil chemical characteristics and suppressed the soilborne plant pathogen *F. oxysporum*. Compost also increased bacterial abundance and altered the taxonomic composition of bacterial communities and abundance of individual bacterial taxa. Functional analysis showed this soil amendment consistently decreased bacterial traits related to carbohydrate use and increased bacteria with the capacity for active microbial dispersal. Outcomes from this research provide novel insight into the impacts of compost on bacterial communities as primary components of soil microbiomes in agricultural ecosystems.

To quantify the effect of green waste compost on soil bacterial communities, bacterial abundance, diversity, and richness were measured. In the greenhouse experiment, 3 weeks after compost application, abundance was slightly increased but only after 8 weeks did 10% compost significantly increase bacterial abundance. Other studies report an increase in microbial biomass, so it was surprising to see that bacterial abundance in the growth chamber experiments did not respond to 10% compost (Table 3) [23, 24]. Bacterial diversity and richness also proved to be unpredictable. Green waste compost application did not change diversity and richness in both greenhouse timepoints, and the second growth chamber experiment but did significantly increase these indices in the first growth chamber experiment. However, our mixed results reflect the lack of consensus, with some green waste compost studies reporting increased diversity and richness and others not finding any difference post application [25, 26].

Of the three most often measured soil chemical characteristics (N, P, K), the addition of green waste compost significantly increased the concentrations of K and P but had no impact on N concentration. This was puzzling because frequently compost increases soil nitrogen content [24, 25, 47]. However, compost rarely does not significantly increase nitrogen concentration, so our results are not vastly different from others [12, 23]. Green waste compost often increases soil K and P concentrations [24–26]. This may be from the nutrients within the compost and the further breakdown of plant or animal material following compost amendment. Soil pH was also altered by green waste compost, which was slightly higher following 5% compost addition and significantly higher when 10% compost had been added. While another study also reports increased pH, green waste compost can also reduce pH [23, 26, 47]. Even within the same study, pH increased in one field and decreased in another following compost application, underlining an overall inconsistency in the response of pH to compost [24]. Unlike pH, soil OM reacts extremely reliably following green waste compost application. The increase in OM in this experiment and all other studies makes sense since compost is made of organic materials that are in various stages of decomposition [24, 47]. Ultimately, green waste compost has potential to change soil chemical characteristics in ways that are still being elucidated.

In agreement with this study, prior research has also found that green waste compost application significantly reduces the abundance of *F. oxysporum* in bulk and rhizosphere soil [20]. One explanation for compost-based suppression of soilborne pathogens like *F. oxysporum* is that it introduces or supports the growth of microorganisms that directly suppress the pathogen [21, 22]. Some of these microorganisms may be responsible for the differences in bacterial community composition observed in compost and control soil. From a taxonomic perspective, this study identified 16 bacterial genera that consistently increased following compost application and could have contributed to the reduction in *F. oxysporum*. For example, the genus *Ureibacillus* that increased from compost in this research was also identified as a primary taxon associated with compost-based suppression of the oomycete plant pathogen *Pythium ultimum* [48]. Interestingly, *Ureibacillus* and other closely related firmicute taxa that showed consistent positive responses to compost in this study, such as *Geobacillus* and *Thermobacillus*, have been identified as playing a role during composting of plant or other substrates [49, 50]. This raises the question of whether compost itself served as a vector to introduce these putative pathogen-suppressing microbial taxa into soil [22, 26, 51].

Bacterial functions related to carbohydrate use were more common in control soil compared to soil amended with compost. One explanation for this result is that the ability of bacteria to use certain carbohydrates may be more important in nutrient poor control soils where compost was not added [52]. For example, glycogen storage and metabolism in some bacteria functions as a reserve source of carbon and energy under starvation conditions [53, 54]. Bacteria with genes similar to the recently described widespread colonization island (WCI) were also more common in control soil. The WCI has been found in bacteria and archaea and harbors genes for a secretion system similar the Type 2 Secretion System and is known to play a role in bacterial biofilm formation and host colonization [55, 56]. Overall, these results suggest that bacterial functions related to gathering carbohydrate resources and protein secretion are more common in non-amended control soils.

Compost consistently increased bacteria predicted to have traits related to motility and chemotaxis, specifically the presence of flagella. The capacity to respond to and move toward or away from chemical gradients is common in many different bacteria [57–59]. Interestingly, these traits may be especially important in mediating interactions between bacteria in soil and plants, including response to root exudates and colonization of plant roots by beneficial bacteria [60–62]. There is some evidence that chemotaxis can also mediate microbial interactions, which could play a role in bacterial suppression of *F. oxysporum* [63, 64]. Recent research has also found that application of green waste compost increases abundance of genes in soil that play a role in microbial chemotaxis [65]. Together, these results highlight the potential for compost to increase bacteria in soil with improved capacity to colonize the rhizosphere or rhizoplane and directly interact with plants.

This study has technical implications for future research focused on linking soil microbiome characteristics with pathogen suppression or other processes relevant to agriculture. All experiments showed that compost application reduced the abundance of the pathogen *F. oxysporum* in soil. This result is consistent with prior research and demonstrates that the suppressive effect of compost on *F. oxysporum* is reproducible across experiments and studies [20]. However, the majority of bacterial functions that were significantly different between control and compost amended soil were not reproducibly affected across the different experiments and environments, despite consistent suppression of *F. oxysporum*. Though rare, a few taxa and functions also showed inverse responses to compost application in different environments. Part of this inconsistency is likely due to the small sample size in the study, which is one of its limitations, or the differences in temperature between greenhouse and growth chamber experiments. Regardless, had we not included replicated experiments we would have over interpreted the number of microbial responses to compost application and their potential role in pathogen suppression. These inconsistencies demonstrate a broader need to encourage within study replicated experiments in microbial ecology research to ensure results are reproducible [66].

This research provides novel insight into the taxonomic and functional responses of soil microbiomes to compost in agricultural soil infested with a common soilborne plant pathogen. The observed functional changes provide additional evidence that compost enriches bacteria with increased active dispersal capacity and decreases bacterial functions tied to carbohydrate use. This increased dispersal capacity may have downstream effects on bacterial colonization of plant roots and potential interactions between bacteria and other microorganisms in soil like the plant pathogens *F. oxysporum*, though this has to be validated. Ongoing field research is evaluating if compost effects bacteria in rhizosphere soil, whether this is predictive of pathogen suppression at the field scale, and if compost serves as a vector for introducing pathogen suppressive bacteria into soil. Ultimately, a better understanding of the effects of compost on soil bacterial communities will improve our ability to manage the microbial mechanisms underlying the beneficial effects of compost on crop and soil health.

## Supplementary Information

Below is the link to the electronic supplementary material.

Supplementary file1 (JPEG 836 KB)

Supplementary file2 (JPEG 1508 KB)

Supplementary file3 (JPEG 650 KB)

Supplementary file4 (JPEG 2529 KB)

Supplementary file5 (XLSX 77 KB)

## Data Availability

Sequence data generated in this study are deposited in the NCBI Sequence Read Archive (SRA) under BioProject PRJNA939197.
