# Supplementary figures and images for: Green Waste Compost Impacts Microbial Functions Related to Carbohydrate Use and Active Dispersal in Plant Pathogen-Infested Soil
Source: Microb Ecol. 2024 Feb 17;87(1):44. doi: 10.1007/s00248-024-02361-8 (PMC10874327; doi:10.1007/s00248-024-02361-8)

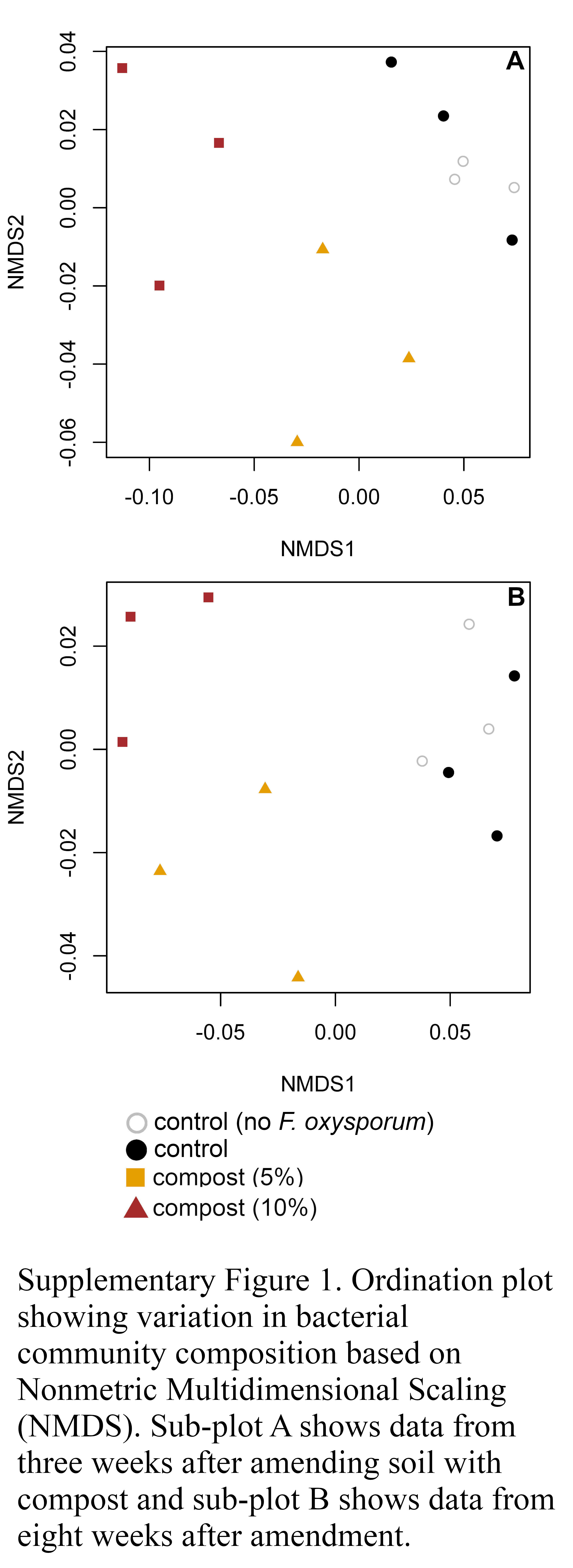

Supplement: Supplementary file 1 — Supplementary file1 (JPEG 836 KB) [file 248_2024_2361_MOESM1_ESM.jpeg]

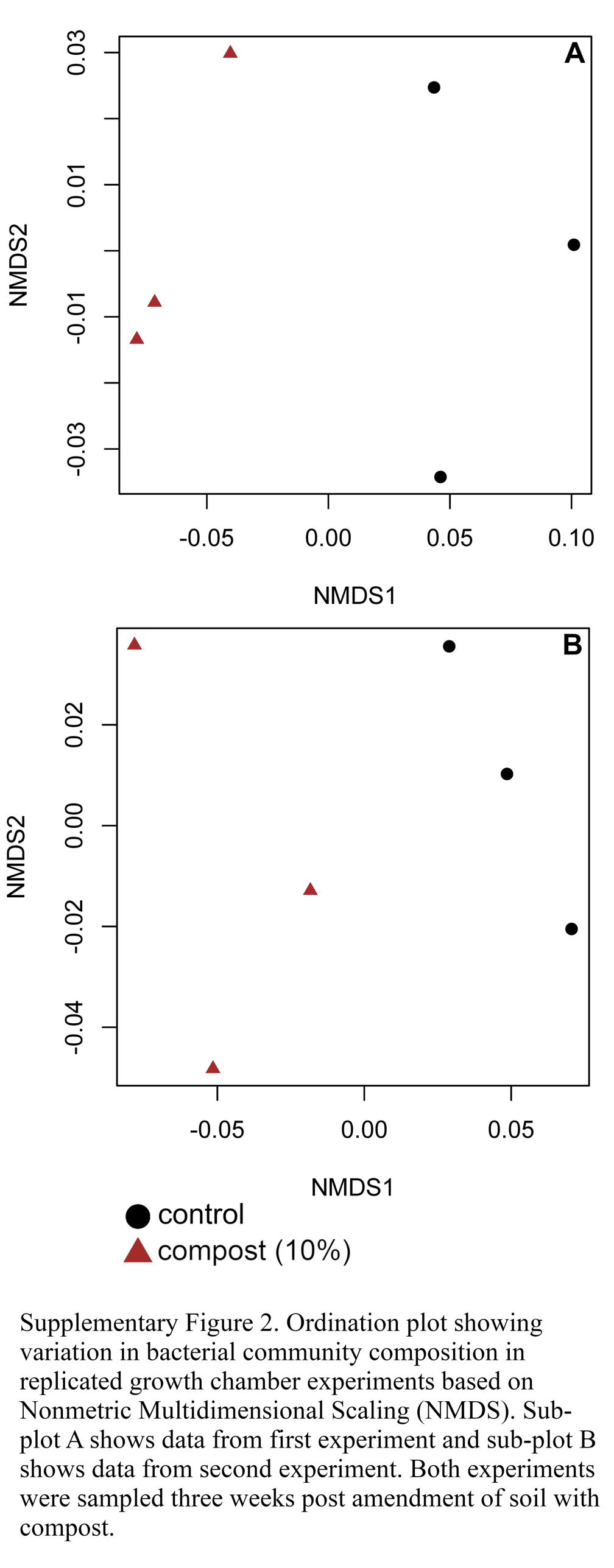

Supplement: Supplementary file 2 — Supplementary file2 (JPEG 1508 KB) [file 248_2024_2361_MOESM2_ESM.jpeg]

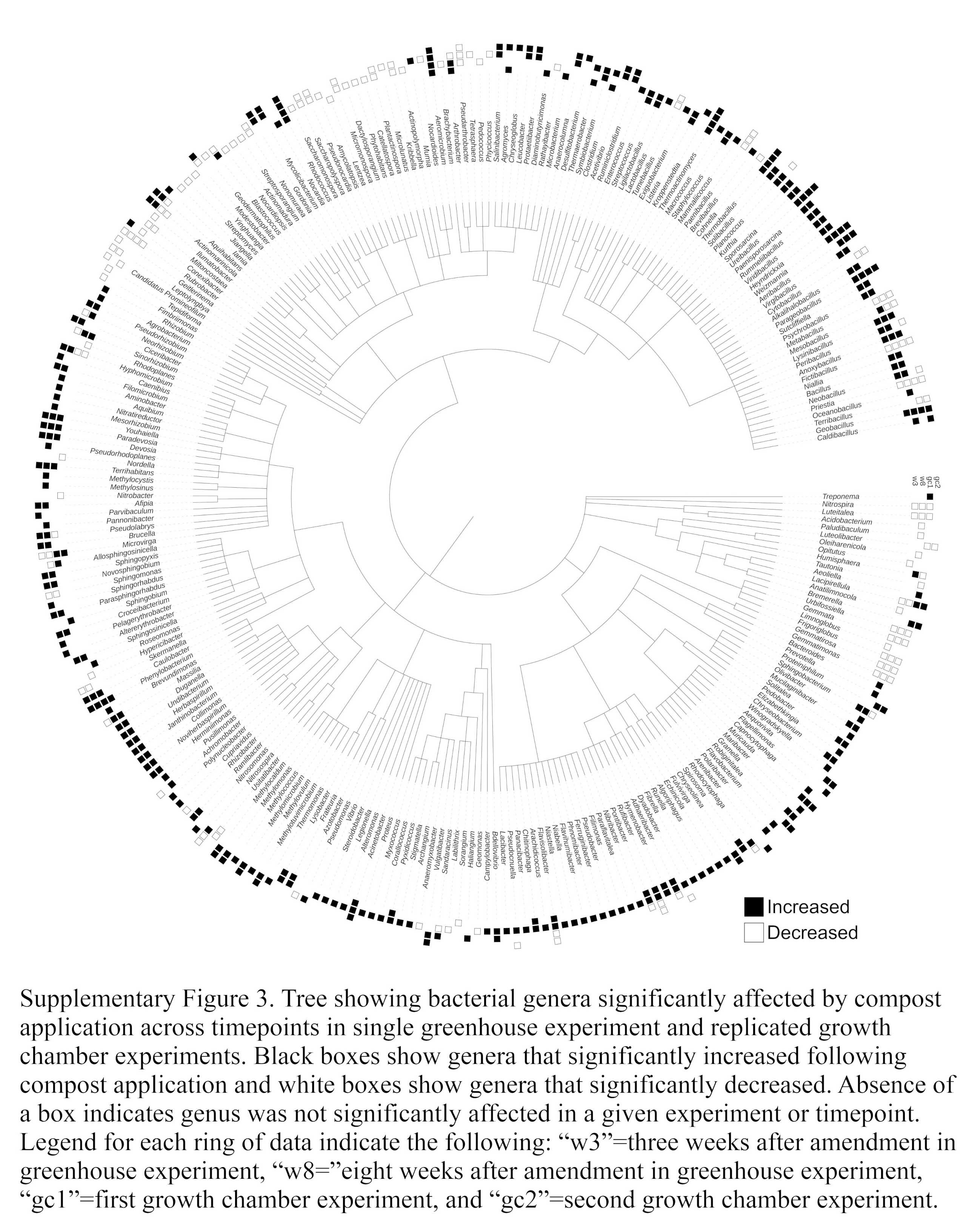

Supplement: Supplementary file 3 — Supplementary file3 (JPEG 650 KB) [file 248_2024_2361_MOESM3_ESM.jpeg]
